# Response to Thalamic Ventralis Intermedius Nucleus Deep Brain Stimulation in Essential Tremor vs. Essential Tremor-Plus

**DOI:** 10.3389/fneur.2021.790027

**Published:** 2021-11-29

**Authors:** Gabriela S. Gilmour, Davide Martino, Karen Hunka, Pia Lawrence, Zelma H. T. Kiss, Veronica Bruno

**Affiliations:** ^1^Department of Clinical Neurosciences, Cumming School of Medicine, University of Calgary, Calgary, AB, Canada; ^2^Hotchkiss Brain Institute, University of Calgary, Calgary, AB, Canada

**Keywords:** essential tremor, tremor, deep brain stimulation, outcome, predictors

## Abstract

**Introduction:** Essential tremor (ET) is a tremor syndrome characterized by bilateral, upper limb action tremor. Essential tremor-plus (ET-plus) describes ET patients with additional neurologic signs. It is unknown whether there is a difference in response to treatment with ventralis intermedius nucleus deep brain stimulation (VIM DBS) in patients with ET and ET-plus. Due to potential variability in underlying etiology in ET-plus, there is a concern that ET-plus patients may have worse outcomes. The aim of this study was to identify whether patients with ET-plus have worse tremor outcomes after VIM DBS than patients with ET.

**Methods:** This is a retrospective chart and video review evaluating VIM DBS outcomes by comparing changes from baseline in the Fahn-Tolosa-Marin Tremor Rating Scale Part B (FTM-B) for the treated limb between patients with ET and ET-plus at follow-up examinations. Patients were re-classified as having ET or ET-plus using pre-operative examination videos by two independent movement disorders neurologists blinded to patient characteristics. As a secondary outcome, we evaluated for correlations and potential predictors of treatment response.

**Results:** Twenty-six patients were included: 13 with ET, 13 with ET-plus. There were no significant differences in the change in FTM-B scores between the ET and ET-plus patients at each follow-up examination. None of the included patients developed new symptoms compatible with dystonia, parkinsonism or gait disturbances.

**Conclusions:** Patients with ET-plus had tremor improvement from VIM DBS, with no differences when compared to those with ET, without emergence of postoperative neurological issues. Patients with ET-plus should still be considered good candidates for VIM DBS for treatment of tremor.

## Introduction

Essential tremor (ET) is a tremor syndrome, classically characterized by bilateral, upper limb, postural or action tremor, which may include tremor in other locations (1). There has been variability in terms of the diagnostic criteria used by clinicians and whether it must be diagnosed in isolation, without other neurologic signs, or may encompass accompanying clinical features. In relation to this, it has been proposed that ET may have multiple underlying etiologies, leading to the multiple observed phenotypes (2). In 2018, a new classification for tremor syndromes was introduced which addressed the phenotypic heterogeneity of ET and other tremor syndromes (3). In this classification, patients with tremor consistent with ET, but with additional neurologic signs of uncertain significance such as impaired tandem gait or dystonia, are classified now as essential tremor-plus (ET-plus).

When severe, ET may lead to social isolation and disability, increasing vulnerability to anxiety and depression (4–6). Currently, no curative treatments exist for ET, but several medications may be used to improve symptoms, including primidone, propranolol, and topiramate (1, 7). Yet, 25–50% of patients do not respond to medications, and many experience significant side effects (1). There also exist several invasive treatments, including lesioning or stimulating the ventralis intermedius nucleus (VIM) of the thalamus, effectively reducing upper extremity tremor (8, 9). In particular, deep brain stimulation (DBS) has been the preferred treatment for patients with refractory ET for the past 2 decades (10). However, there are some risks associated with DBS, which must be considered when determining whether a patient may benefit from it, including paresthesias, headache, gait disorder, dysarthria, stroke, and death (7).

DBS provides both symptomatic and functional benefits to patients with ET very early in the postoperative period (11). An improvement in tremor severity by 40% has been observed, which persists with long-term follow-up (12). Studies of long-term outcomes from VIM DBS for ET have reported progressive reduction in efficacy in 16–73% of patients, with mean follow-up ranging from 1–5 years (13–16). Given the new classification for tremor, it is unknown whether there is a difference in treatment response in patients with ET and ET-plus. Because of the potential variability in underlying etiology in ET-plus, it raises the concern that ET-plus patients may have worse long-term outcomes.

The primary objective of this study was to identify whether patients with ET-plus have worse long-term tremor outcomes after VIM DBS than patients with ET. We hypothesized that patients with ET-plus would have worse tremor outcomes than the pure ET patient group. Additionally, we aimed to explore demographic or clinical factors in patients with both ET and ET-plus that could predict loss of benefit with VIM DBS over time.

## Materials and Methods

We conducted a retrospective chart and video review of all patients who underwent VIM DBS for ET and ET-plus at the Foothills Medical Center in Calgary, Alberta, between 2001 and 2018. ET was defined based on the tremor classification as “isolated tremor syndrome of bilateral upper limb action tremor, at least 3 years' duration, with or without tremor in other locations (e.g. head, voice or lower limbs), and the absence of other neurological signs, such as dystonia, ataxia or parkinsonism” (3). ET-plus was defined as “tremor with the characteristics of ET and additional neurological signs of uncertain significance such as impaired tandem gait, questionable dystonic posturing, memory impairment, or other mild neurological signs of unknown significance that do not suffice to make an additional syndrome classification or diagnosis” (3). Inclusion criteria were: (1) clinical diagnosis of ET or ET-plus at the time of DBS implantation, agreed upon by two movement disorder neurologists and one functional/stereotactic neurosurgeon; (2) DBS insertion in the VIM nucleus of the thalamus; (3) at least one-year follow-up duration; (4) age ≥ 18-years-old. Exclusion criteria included missing tremor severity data at baseline or postoperative clinical assessment, and surgical complications resulting in failed implantation or removal of device.

Data from the preoperative baseline and at each postoperative follow-up were extracted from the charts. Baseline clinical characteristics collected included: gender, handedness, age of onset of ET, age at surgery, family history (tremor, Parkinson Disease [PD], developmental delay), tremor characteristics (body distribution, activation conditions, symmetry, improvement with alcohol), previous treatments including maximum dose (propranolol, primidone, topiramate, clonazepam, gabapentin), additional neurological signs of uncertain significance (dystonia, rigidity, bradykinesia, myoclonus, mild cognitive impairment identified during presurgical neurocognitive assessment, impaired tandem gait) and etiology (acquired, genetically defined, idiopathic familial or idiopathic sporadic). Impaired tandem gait was defined as greater than one out-of-line misstep on a 10-step tandem gait (17). Bradykinesia was evaluated using finger tapping, hand movements, pronation-supination movements, toe tapping and leg agility, with abnormality defined as any of the following: regular rhythm is broken by interruptions or hesitations of movement, slowing of movements, amplitude decrement. Occasionally, when patients have severe action tremor, tremor interferes with the ability to adequately rest the arms for assessment of rest tremor. In these cases, patients were not classified as clearly having rest tremor. Tremor characteristics to appropriately classify patients as having ET or ET-plus were extracted independently from pre-operative videos by two movement disorders specialists (VB and DM), blinded to patient characteristics, with supplementation from paper charts for aspects of the examination not filmed (e.g. mild cognitive impairment). As emphasized by the consensus statement on the classification of tremor, the interpretation of these soft neurological signs is subjective (3), but consensus was achieved by both movement disorders specialists for the interpretation of soft signs and classification given as part of the present study. Postoperative videos and chart notes from follow-up visits were used to detect new-onset additional neurological findings such as the development of dystonia, gait disturbances or parkinsonian manifestations after surgery. Using the baseline chart and video data, the tremor was classified to Axis 1 tremor syndromes as ET or ET-plus, as described by the Task Force on Tremor of the International Parkinson and Movement Disorder Society (3). Structural imaging was reviewed when available for evidence of small vessel disease, stroke, basal ganglia abnormalities, and other intracranial abnormalities.

Surgery was performed by one surgeon (ZK) and utilized magnetic resonance-guided stereotaxy based on coordinates of the anterior and posterior commissure and the Schaltenbrand and Wahren brain atlas (18). Intraoperative microelectrode recordings and micro-stimulation were used to optimize anatomical targeting and correct placement confirmed with postoperative magnetic resonance imaging. Electrodes were implanted and externalized for several days to allow for postoperative testing for tremor effects and side-effects prior to a second surgical procedure in which the pulse generator was implanted. All patients were implanted with Medtronic devices.

Patients were seen in follow-up 6 months postoperatively and then on an annual basis. Tremor severity data using the Fahn-Tolosa-Marin Tremor Rating Scale (FTM) (19) was recorded prospectively in a database by movement disorder nurse clinicians (KH and PL) at each follow-up appointment. Stimulation parameter data (contact configuration, voltage, pulse width, frequency) were extracted from the records from the first and most recent programming session.

Tremor outcomes were assessed using the FTM (19). FTM is divided into three parts: (A) quantifies the tremor at rest, with posture and with action in 9 body parts; (B) rates action tremor of the upper extremities (FTM-B); and (C) assesses functional disability, with higher scores indicating worse disability (19). As primary outcome, changes in FTM-B scores for the treated limb were compared between ET and ET-plus at 1 year, 2 years, 3–5 years and 6-10 years follow-up. FTM-B was selected due to its specificity for action tremor of the upper limb, given the aim of DBS for ET is typically to reduce upper limb tremor (19). For patients with bilateral staged treatments we considered only the first treated side and included only the data obtained before the second implant, as has been done in previous studies (20). Additionally, we explored associations between worse FTM-B outcomes and demographic/clinical variables at each follow-up visit, searching for potentials predictors of response.

The study was approved by the Conjoint Health Research Ethics Board, University of Calgary (REB18-2052, approved January 21, 2019).

### Statistical Analysis

The main goal was to compare VIM DBS outcomes between ET and ET-plus groups by comparing changes from baseline in the FTM-B scores of the treated limb at each follow-up examination at 1 year, 2 years, 3–5 years and 6–10 years. All analyses were performed for the entire study population and subdivided by underlying diagnosis of ET or ET-plus. FTM-B scores were subdivided into scores for the limbs contralateral to the DBS device and recorded as absolute values. The change from baseline at each subsequent time-point was also calculated. Baseline quantitative variables were analyzed using *t*-tests and categorical data using Wilcoxon Rank Sum Tests. Values are expressed as mean ± standard deviation. A *p*-value < 0.05 was considered statistically significant.

Wilcoxon Signed Rank comparisons for non-parametric data were used to compare tremor scores for baseline and follow-up. Additionally, a Friedman test was used to compare performance at all follow-up examinations and assess potential change in tremor within the follow-up period. Spearman's correlation and point-biserial correlation coefficient were used to explore associations between continuous and categorical variables respectively, and changes in FTM-B in the different follow-up visits. The type I error rates for multiple comparisons were corrected with Holm-Bonferroni.

Linear regression models were built to identify predictors of treatment response, with the dependent variable being the changes in the FTM-B scores from baseline to the different follow-up times in the entire study population. Independent variables were selected according to the results of the bivariate correlations, as well as introducing those variables that could be confounders or effect modifiers, such as age and gender. The models with higher R squared values were reported. All relevant variables were tested for multicollinearity.

To confirm sufficient power in our analysis, we calculated that a total sample size of 18–20 was adequate to detect differences of at least 6 points (SD 4) in the FTM-B scores between ET and ET-plus with a power >90% (alpha 0.05) using two independent means test. We used the FTM-B 6 ± 4 points score change based on the results from previous studies (12, 21). There is no definite cut-off to define minimal significant differences when using the FTM tremor scale (12, 21). All analyses were performed using Stata SEv16.0.

## Results

Charts were reviewed for 35 patients undergoing VIM DBS implantation. A total of 9 patients were excluded due to: surgical complication affecting outcome (*n* = 2), and missing FTM-B data from baseline or post-surgical visits (*n* = 7). In total, 26 patients were included in the final analysis.

### General Clinical and Tremor Characteristics

Seventeen patients were male (65.4%), and the age at surgery was 65.0±8.0 years. General clinical and tremor characteristics at baseline are found in Table 1. Detailed stimulation parameters are found in Supplementary Table 1. Magnetic resonance imaging was available for 23 patients (88.5%), which demonstrated small vessel disease in 19, evidence of previous ischemic stroke in 3, arachnoid cyst in middle cranial fossa in 1 and mild generalized cerebral atrophy in 1. None of the imaging revealed basal ganglia pathology.

**Table 1:** ET vs. ET-plus clinical characteristics.

	**Total** ***n* = 26**	**ET** ***n* = 13**	**ET-plus ** ***n* = 13**	**ET vs. ET-plus** ***p*-value**
Male gender	17 (65.4%)	9 (69.2%)	8 (61.5%)	0.68
Age at surgery (years)	65 ± 8.0	62.2 ± 6.3	67.8 ± 8.9	0.07
Age onset (years)	34.3 ± 17.8	35.8 ± 15.6	32.8 ± 20.2	0.66
Onset to surgery (years)	30.7 ± 17.5	26.3 ± 15.8	35.1 ± 18.7	0.20
Left-hand dominant	4 (15.4%)	2 (15.4%)	2 (15.4%)	1.00
**Medication trials**				
Propranolol	21 (80.8%)	11 (84.6%)	10 (76.9%)	0.62
Primidone	23 (88.5%)	12 (92.3%)	11 (84.6%)	0.54
Topiramate	24 (92.3%)	12 (92.3%)	12 (92.3%)	1.00
Clonazepam	3 (11.5%)	2 (15.4%)	1 (7.7%)	0.54
Gabapentin	12 (46.1%)	7 (53.8%)	5 (38.5%)	0.44
**Alcohol response**	8 (30.7%)	5 (38.5%)	3 (23.1%)	NA
**Family history**				
ET	16 (61.5%)	8 (61.5%)	8 (61.5%)	1.00
PD	4 (15.4%)	3 (23.1%)	1 (7.7%)	NA
**Tremor location**				
Both hands	25 (96.1%)	13 (100%)	12 (92.3%)	0.31
One hand	1 (3.8%)	0 (0%)	1 (7.7%)	NA
Head/neck	20 (76.9%)	7 (53.8%)	13 (100%)	<0.05^*^
Voice	9 (34.62%)	4 (30.7%)	5 (38.5%)	0.68
Jaw/lips/tongue	4 (15.4%)	0 (0%)	4 (30.8%)	0.03^*^
Both legs	4 (15.4%)	2 (15.4%)	2 (15.4%)	1.00
One leg	2 (7.7%)	0 (0%)	2 (15.4%)	0.15
**Body distribution**				
Segmental	18 (69.3%)	9 (69.3%)	9 (69.3%)	1.00
Generalized	8 (30.8%)	4 (30.8%)	4 (30.8%)	1.00
**Activation conditions**				
Rest	4 (15.4%)	0 (0%)	4 (30.8%)	0.03^*^
Postural	26 (100%)	13 (100%)	13 (100%)	1.00
Kinetic simple	24 (92.3%)	11 (84.6%)	13 (100%)	0.15
Kinetic intention	8 (30.8%)	3 (23.1%)	5 (38.5%)	0.40
**Additional subtle features**				
Dystonia	7 (26.9%)	0 (0%)	7 (53.9%)	<0.05^*^
Rigidity	2 (2.7%)	0 (0%)	2 (15.4%)	0.15
Bradykinesia	4 (15.4%)	0 (0%)	4 (30.8%)	0.03^*^
Myoclonus	1 (3.8%)	0 (0%)	1 (7.7%)	0.31
Mild cognitive impairment	2 (7.7%)	0 (0%)	2 (15.4%)	0.15
Impaired tandem	1 (3.8%)	0 (0%)	1 (7.7%)	0.31
Subtle body posture	1 (3.8%)	0 (0%)	1 (7.7%)	0.31
**Baseline characteristics**				
Total FTM scores	51.8 ± 14.1	50.7 ± 14.2	52.9 ± 14.5	0.97
Treated side FTM-B scores	12.1 ± 3.5	12.0 ± 3.7	12.1 ± 3.4	0.95
SF-36	106.5 ± 17.7	109.9 ± 10.5	22.1 ± 22.1	0.75
Beck's Depression Inventory-II	7.8 ± 5.7	7.8 ± 5.9	7.8 ± 5.9	0.89

*Baseline clinical and tremor characteristics of all patients and comparison between those with ET and ET-plus*.

*Continuous values are means ± standard deviation. Categorical data are n (percentage)*.

* *p-value < 0.05. (ET, essential tremor; ET-plus, essential tremor plus; FTM, Fahn-Tolosa-Marin Tremor Rating Scale; FTM-B, Fahn-Tolosa-Marin Tremor Rating Scale-Part B; NA, not applicable; PD, Parkinson Disease; SF-36, 36-Item Short Form Survey)*.

### General Treatment Outcomes

Twenty-two patients underwent unilateral DBS, and 4 patients underwent bilateral implantation. Mean follow-up is 57 ± 43.5 months, with a range of follow-up from 12 months to 15 years.

Two patients required temporary removal of hardware with later re-implantation: one from delayed erosion at implanted pulse generator site 3 years postoperatively and another from erosion at scalp insertion site 1 year postoperatively. One patient had an early superficial infection managed with antibiotics. One patient had a small intraventricular hemorrhage during implantation that did not influence the outcome.

A total of 46 videos were reviewed. Twenty-two patients had preoperative videos, and 24 had postoperative follow-up videos available. Of the postoperative videos, 20 (83.3%) were recorded in the first year of follow-up. Two were recorded at Year 2 follow-up and 2 at Year 3–5 follow-up. For those patients with longer postoperative times, comprehensive follow-up notes were reviewed in detail. For those patients whose preoperative videos could not be obtained, neurologist, neurosurgeon and specialized nursing notes were available. Three patients had ET with no mention of any additional signs or symptoms, and one showed questionable mild hand dystonia present in the preoperative visits. The two patients without postoperative videos had ET without other features before surgery. They did not develop new symptoms after surgery, based on chart notes.

None of the patients in either ET or ET-plus group developed a new onset of non-tremor neurological findings, such as dystonia or parkinsonism, postoperatively. None of the patients with dystonic features at baseline showed significant worsening of those symptoms after DBS implantation.

### ET vs. ET-Plus

Upon review of videos and charts from baseline assessment, of the 26 patients, 13 met a diagnosis of ET (50.0%), and 13 of ET-plus (50.0%). Of those with ET-plus, 7 had signs of subtle or questionable dystonic posturing (1 associated with mild bradykinesia and impaired tandem gait, 1 with mild cognitive impairment), 3 had questionable bradykinesia (1 with mild rigidity), 1 had mild rigidity, 1 had mild cognitive impairment and 1 had questionable myoclonus.

The comparison between ET and ET-plus patients revealed no significant differences, except for the presence of additional neurological signs or tremor at rest, as would be expected based on their clinical definition (Table 1). Additionally, ET-plus patients were more likely to show head/neck tremor (100 vs. 53.8%, *p* ≤ 0.01) and/or tremor of the jaw, lips or tongue (30.8 vs. 0%, *p* = 0.03).

The Friedman test (repetitive measures), conducted to examine the change in FTM-B in each follow-up, showed no statistically significant differences in the FTM-B scores between follow-ups between patients (Q(3) = 6.14, *p* = 0.10), or when stratifying by ET or ET-plus (Q(3) = 6.57, *p* = 0.09).

**Figure 1:**
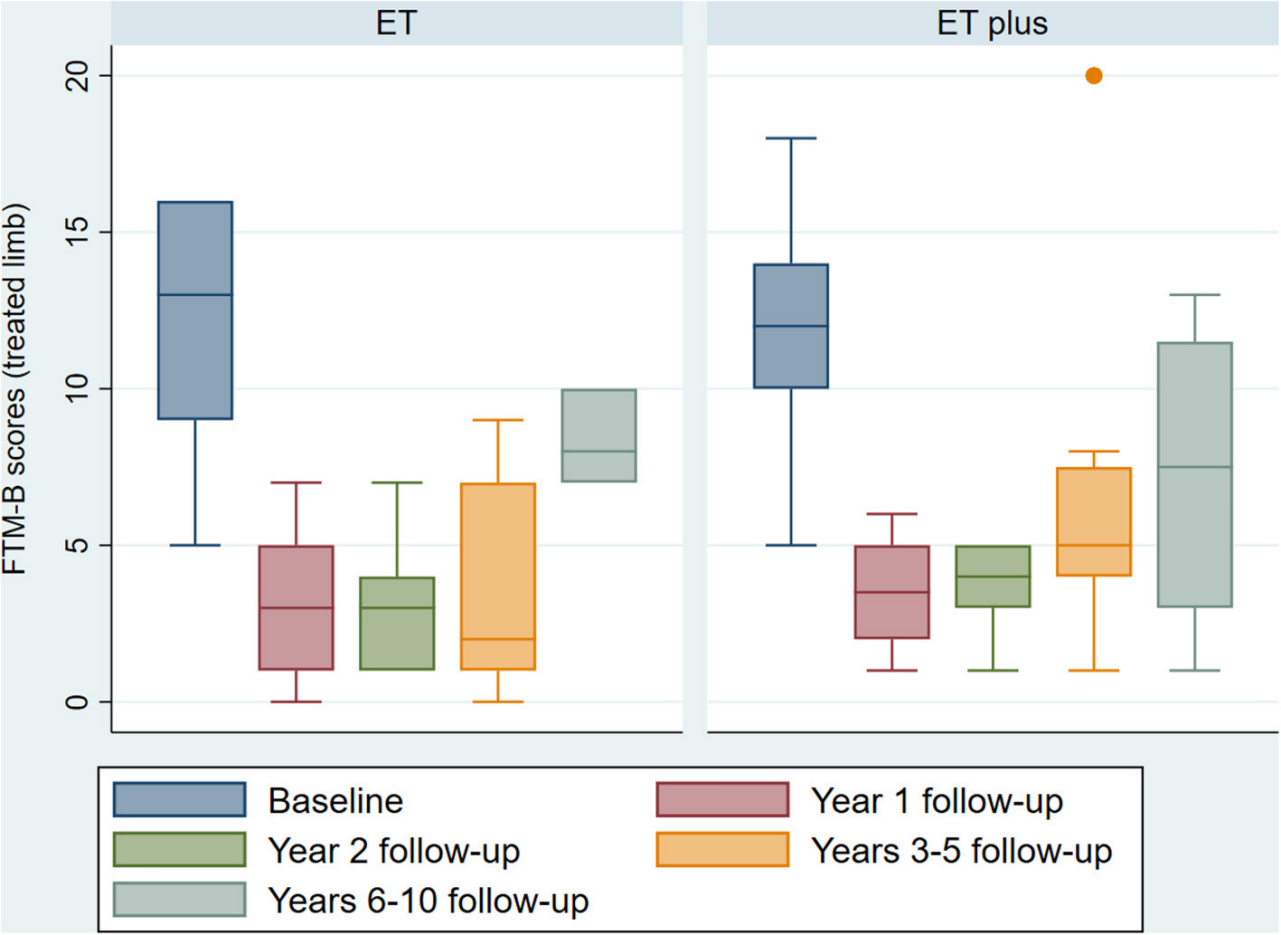
ET vs. ET-plus outcomes. Boxplot of baseline and follow-up FTM-B scores at 1, 2, 3–5 and 6–10 years for the treated limbs of ET and ET-plus patients. One patient in the ET plus group lost benefit from DBS at 3–5 years, shown as the outlier. (ET, essential tremor; ET plus, essential tremor plus; FTM-B, Fahn-Tolosa-Marin Tremor Rating Scale-Part B).

Figure 1 shows that there was no significant difference seen in baseline tremor severity between ET and ET-plus, measured with FTM-B for the treated limb (ET 12.0 ± 3.7 points vs. ET-plus 12.1 ± 3.4 points, *p* = 0.95). There was also no significant difference in the change in FTM-B scores for the treated limbs between the ET and ET-plus patients at each of the follow-up time points (Table 2). Splitting the cohort based on left vs. right implantation of VIM DBS did not reveal any statistically significant differences between tremor response between ET and ET-plus at each time point. Of note, postoperative follow-up time points had different sample sizes due to recent DBS surgery limiting duration of follow-up, missed appointments, and loss to follow-up.

**Table 2:** ET vs. ET-plus outcomes.

**FTM-B tremor severity (change from baseline)**	**Total**	**ET**	**ET-plus**	**ET vs. ET-plus** ***p*-value**
Year 1 follow-up	−9.3 ± 3.6 (*n* = 20)	–9.7 ± 4.2 (*n* = 10)	–9.0 ± 3.1 (*n* = 10)	0.59
Year 2 follow-up	–9.3 ± 3.1 (*n =* 18)	–10.2 ± 3.3 (*n =* 9)	–8.4 ± 2.6 (*n =* 9)	0.12
Years 3–5 follow-up	-7.5 ± 3.6 (*n =* 15)	–9.6 ± 4.1 (*n =* 7)	-5.7 ± 5.4 (*n =* 8)	0.16
Years 6–10 follow-up	-3.7 ± 5.6 (*n =* 7)	–3 ± 5 (*n =* 3)	–4.2 ± 6.8 (*n =* 4)	0.72

*Comparison of change in tremor severity with FTM-B scores for the treated limbs in patients with ET vs. ET-plus at each follow-up time point*.

*Continuous values are means ± standard deviation. (ET, essential tremor; ET-plus, essential tremor plus; FTM-B, Fahn-Tolosa-Marin Tremor Rating Scale-Part B)*.

**Figure 2:**
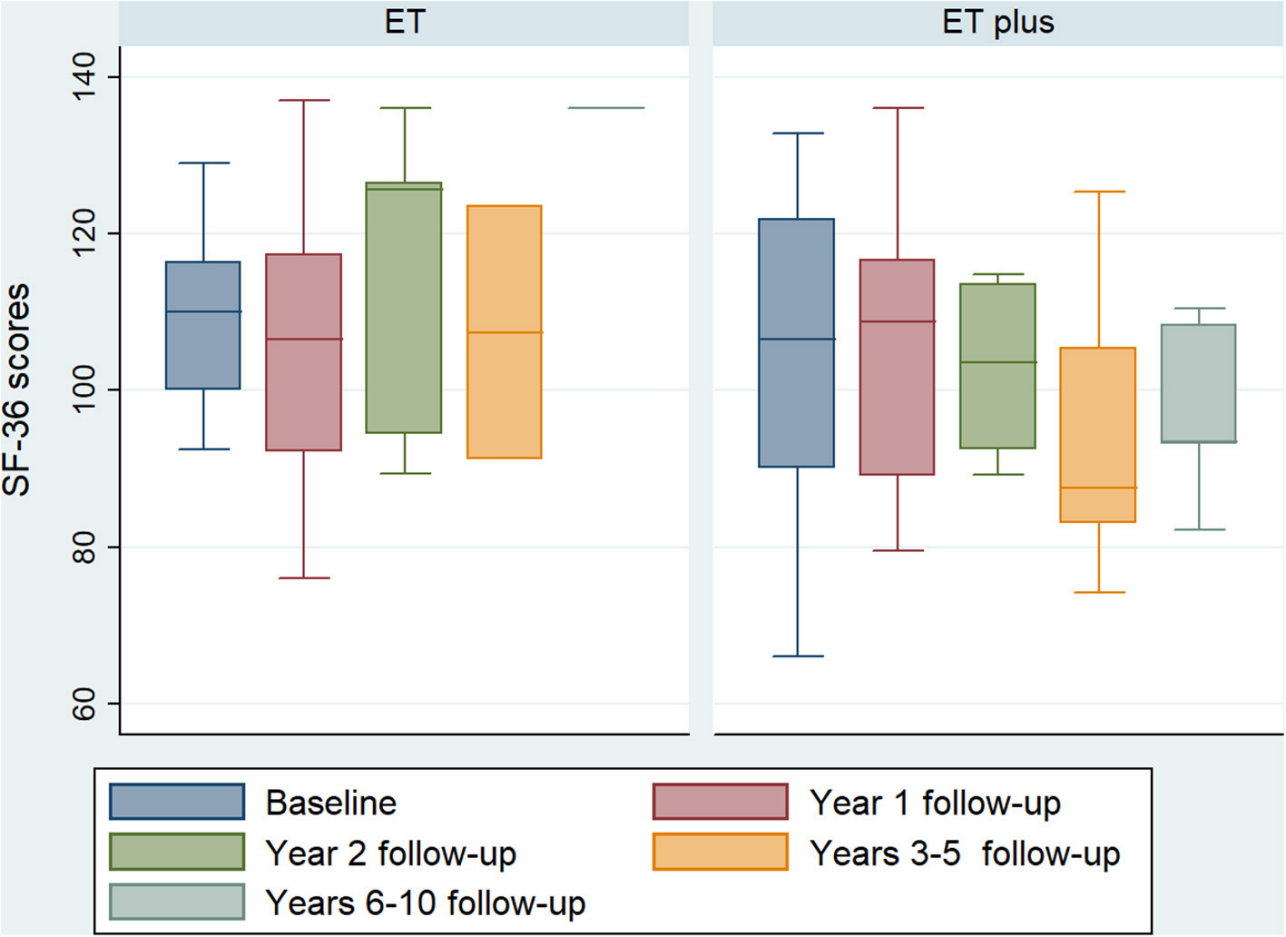
ET vs. ET-plus quality of life outcomes. Boxplot of baseline and follow-up SF-36 scores at 1, 2, 3–5 and 6–10 years follow-up of ET and ET-plus patients. There are no significant differences between ET and ET-plus outcomes at each time point. (ET, essential tremor; ET-plus, essential tremor plus; SF-36, 36-Item Short Form Survey).

Quality of life was measured using the 36-Item Short Form Survey (SF-36) and compared at each follow-up interval between ET and ET-plus patients. There were no statistically significant differences of SF-36 score between ET and ET-plus treated patients at each time point (Figure 2).

### Predictors of Treatment Response

In the Spearman correlation analysis, absolute FTM-B scores at baseline for the treated limb showed correlation with postoperative changes in FTM-B scores at Year 1 (*r* = 0.60, *p* = 0.01) and Year 2 (*r* = 0.78, *p* < 0.01). Both correlations remained significant after adjusting for multiple comparisons (Year 1: *r* = 0.60, *p* = 0.05; Year 2: *r* = 0.78, *p* < 0.01). The point biserial analysis for categorical variables showed that the presence of voice tremor at baseline was negatively correlated with changes in FTM-B scores from baseline at Years 3–5 follow-up (*r* = −0.60, *p* = 0.02). Family history of PD also showed a negative correlation with changes in tremor scores at Year 1 follow-up (*r* = −0.44, *p* = 0.04). Both correlations persisted after Holm-Bonferroni correction for multiple comparisons. Additional explored variables showed no correlation with the primary outcome (Supplementary Tables 2, 3).

To further explore possible predictors of treatment response, we built linear regression models using changes in FTM-B scores of the treated limb at the follow-up times as dependent variables. Those models with higher R-squared (Year 1: R^2^ = 0.82; Year 2: R^2^ = 0.83; Years 3–5: R^2^ = 0.72) are reported (Table 3). The Years 6–10 follow-up model was excluded due to insufficient sample size. The significant relationship between voice tremor at baseline and change from baseline in FTM-B at Years 3–5 follow-up persisted after adjusting for FTM-B at baseline, age at surgery, age at tremor onset, gender, segmental tremor, family history of PD and ET-plus diagnosis (Years 3–5: βr = −10.3, 95% CI −18.94–(−1.61), *p* = 0.03). Among the 9 patients with voice tremor undergoing VIM DBS for treatment of upper limb tremor, 5 had left VIM electrodes, 1 had a right-sided electrode and 2 had bilateral implantations.

**Table 3:** Linear regression model.

**Model R-squared**	**FTM-B change year 1 follow-up**			**FTM-B change year 2 follow-up**			**FTM-B change year 3–5 follow-up**		
	**0.82**			**0.83**			**0.72**		
	**Coef**.	***p*-value**	**95% CI**	**Coef**.	***p*-value**	**95% CI**	**Coef**.	***p*-value**	**95% CI**
FTM-B treated limb at baseline	0.75	0.00^*^	0.47–1.03	0.88	0.01^*^	0.41–1.36	0.69	0.26	−0.67–2.06
Age at surgery	0.00	0.97	−0.19–0.18	0.01	0.88	−0.23–0.26	0.13	0.65	−0.52–0.78
Gender	−1.67	0.29	−4.96–1.62	−0.47	0.79	−4.46–3.50	3.97	0.39	−6.55–14.49
Age at tremor onset	−0.02	0.47	−0.09–0.04	0.02	0.60	−0.06–0.10	−0.02	0.81	−0.23–0.19
Family history of PD	−1.14	0.42	−4.13–1.85	0.83	0.46	−1.63–3.31	2.40	0.47	−5.20–10.00
Voice tremor at baseline	−1.36	0.28	−4.03–1.30	−1.99	0.22	−5.39–1.40	−10.27	0.03^*^	−18.94–(−1.61)
Segmental tremor	−1.27	0.30	−3.85–1.32	−0.42	0.74	−3.20–2.36	0.16	0.96	−7.12–7.44
ET-plus	−0.73	0.49	−2.98–1.52	−0.74	0.55	−3.48–1.98	−1.41	0.66	−8.96–6.14

*Dependent variable: change in FTM-B scores for the treated limb at each follow-up time*.

* *p-value < 0.05. (CI, confidence interval; Coef., coefficient; ET-plus, essential tremor plus; FTM-B, Fahn-Tolosa-Marin Tremor Rating Scale-Part B; PD, Parkinson Disease)*.

Additionally, the correlation between baseline FTM-B in the treated limb and changes in the score at Year 1 follow-up and Year 2 follow-up remained significant in the same linear regression model (Year 1: βr = 0.75, 95% CI 0.47–1.03, *p* < 0.01; Year 2: βr = 0.88, 95% CI 0.41–1.36, *p* = 0.01). The rest of the explored variables showed no significant association with changes in tremor scores from baseline in any of the analyzed follow-ups in the adjusted model.

## Discussion

The main goal of this single-center retrospective study was to identify whether patients with ET-plus experience less improvement in tremor after VIM DBS compared to patients with ET. Our patients with ET-plus did not have worse short- or long-term motor outcomes compared to patients with ET after receiving VIM DBS. Additionally, our patients with ET-plus did not have worse quality of life measures at each follow-up interval compared to patients with ET. This is a reassuring finding, providing evidence for the ongoing use of VIM DBS in ET-plus patients, despite the presence of additional neurological signs. This finding supports recently published research showing that VIM DBS is an effective treatment for ET-plus, with a similar degree of tremor suppression as seen in ET (22).

The evidence for the use of VIM DBS for ET-plus is particularly important in light of recent studies showing clinically relevant dystonia emerging after thalamic neurosurgical procedures (23, 24). In our series, none of the patients developed new symptoms compatible with dystonia, parkinsonism or gait disturbances after the VIM DBS surgery. Thus, in ET-plus with subtle dystonia, our study suggests that VIM DBS should continue to be offered to patients with medically refractory tremor. Currently, there is no published research comparing unilateral thalamotomy in the treatment of essential tremor-plus vs. essential tremor, leaving it as an important area for further evaluation. Given the published cases of clinically relevant dystonia emerging after thalamotomy for treatment of tremor in patients with pre-existing dystonia, caution should be exercised when considering thalamotomy for the treatment of ET-plus, with DBS likely being a safer option (24).

Additionally, we aimed to explore potential predictors of tremor response to VIM DBS over time for patients with ET and ET-plus. Higher baseline FTM-B score was correlated with larger changes in the same limb tremor scores at Year 1 and Year 2 follow-up, as has been shown in previous studies (20). Over time, this difference may become less significant as all patients experience some worsening of tremor (25). Although not statistically significant in the present study, a trend is seen of worsening of tremor severity at the 6–10 years post-operative follow-up interval in both ET and ET-plus groups (Figure 1). Previous studies of long-term outcomes from VIM DBS for ET have described up to 35% experiencing waning of benefit, while other have described up to 70% experiencing loss of benefit (13–16). It appears that this loss of benefit is not mediated by diagnosis of ET vs. ET-plus, but may instead be related to disease progression, misdiagnosis, or tolerance to stimulation (12). Further research must be undertaken to better evaluate for predictors of long-term treatment effect.

The presence of voice tremor at baseline was found to be associated with worse contralateral hand tremor outcomes at Years 3–5 follow-up when patients underwent VIM DBS for treatment of upper limb tremor. Outcomes of voice tremor treatment were not evaluated in this study. This finding may indicate an alternate underlying diagnosis to that of ET or ET-plus, such as segmental dystonia, particularly when the voice tremor is the predominant symptom (3). It should be noted that this does not however indicate that VIM DBS is inappropriate in the treatment of vocal dystonia. A recent small randomized controlled trial has shown left VIM DBS to trend toward improvement in quality of life and voice quality in patients with adductor spasmodic dystonia, a task-specific vocal cord dystonia (26).

There were several demographic, imaging and clinical characteristics, including specific tremor features, evaluated in this study that did not show correlation with post-VIM DBS tremor scores. These included age at surgery, age of onset of tremor, body distribution, and duration of time between the onset of tremor and surgery, as supported by previous research (20). Additionally, alcohol responsiveness did not predict response to DBS, which is in agreement with a previous study (20). A lack of benzodiazepine response has previously been described as a predictor for outcome after DBS, which our study failed to corroborate (20).

This study has a number of limitations. Most importantly, it is retrospective in nature, with small sample size. We did not have access to 3D data regarding electrode location, and thus could not include the location of active contacts in standardized space with statistical analysis comparing ET and ET-plus. In addition, nurse clinicians, when evaluating FTM, were not blinded to patient characteristics or treatment. To overcome limitations from its retrospective nature, we examined the videos blinded to time point, treated limb, and clinical characteristics to extract tremor characteristics and classification. Regardless, this comprehensive study is among the largest studies evaluating VIM DBS specifically for ET and ET-plus using the 2018 classification for tremor, providing evidence for ongoing use of DBS in both ET and ET-plus.

In our small cohort, there are no significant differences in short- and long-term motor treatment response between ET and ET-plus patients treated with VIM DBS. Further prospective research with larger sample sizes are necessary to further understand the predictors of treatment failure in ET and ET-plus, allowing for appropriate patient selection for invasive treatment of tremor. Our findings suggest that patients with ET-plus, resulting in additional neurological signs, should continue to be considered for DBS for treatment of action tremor.

## Data Availability Statement

The raw data supporting the conclusions of this article will be made available by the authors, without undue reservation.

## Ethics Statement

The studies involving human participants were reviewed and approved by Conjoint Health Research Ethics Board, University of Calgary (REB18-2052, approved January 21, 2019). Written informed consent for participation was not required for this study in accordance with the national legislation and the institutional requirements.

## Author Contributions

GG: conceptualization, methodology, investigation, data curation, formal analysis, and writing — original draft. DM: methodology, investigation, and writing — review & editing. KH and PL: resources, investigation, and writing — review & editing. ZK: methodology, resources, investigation, writing — review & editing, and funding acquisition. VB: conceptualization, methodology, investigation, formal analysis, writing — review & editing, supervision, and funding acquisition. All authors read and approved the final manuscript.

## Funding

This work was supported by the Neuromodulation Research & Education Fund, Alberta Health Services and Natural Sciences and Engineering Research Council of Canada (#RGPIN/04126-2017) grant to ZK.

## Conflict of Interest

The authors declare that the research was conducted in the absence of any commercial or financial relationships that could be construed as a potential conflict of interest.

## Publisher's Note

All claims expressed in this article are solely those of the authors and do not necessarily represent those of their affiliated organizations, or those of the publisher, the editors and the reviewers. Any product that may be evaluated in this article, or claim that may be made by its manufacturer, is not guaranteed or endorsed by the publisher.
