# Supplementary material for: Response to Thalamic Ventralis Intermedius Nucleus Deep Brain Stimulation in Essential Tremor vs. Essential Tremor-Plus
Source: Front Neurol. 2021 Nov 29;12:790027. doi: 10.3389/fneur.2021.790027 (PMC8666686; doi:10.3389/fneur.2021.790027)
Supplement: Supplementary file 1 [file Table_1.DOCX]

Supplementary Material

# Supplementary Tables

**Supplementary Table 1.** Initial and final stimulation parameters and patient characteristics. (ET = essential tremor, ET-plus = essential tremor-plus; F = female; M = male; VIM = ventralis intermedius nucleus)

| Patient ID | Sex | Age | Syndrome | Electrode Location | Initial Stimulation Parameters | Final Stimulation Parameters |
| --- | --- | --- | --- | --- | --- | --- |
| 1 | F | 75 | ET-plus | Right VIM | 6-5+, 60μs, 130Hz, 2.0mA | 6-5+, 60μs, 130Hz, 2.2mA |
| 2 | M | 66 | ET | Left VIM | 2-3+, 90μs, 135Hz, 3.0mA | 2-3+, 90μs, 135Hz, 2.6mA |
| 3 | M | 56 | ET | Right VIM | 6-C+, 90μs, 145Hz, 3.2mA | 6-C+, 90μs, 145Hz, 3.6mA |
| 4 | F | 65 | ET | Left VIM | 2-1+, 60μs, 130Hz, 2.6mA | 2-1+, 60μs, 130Hz, 2.9mA |
|  |  | 66 |  | Right VIM | 6-5+, 60μs, 130Hz, 2.0mA | Not Available |
| 5 | M | 61 | ET-plus | Left VIM | 2-C+, 60μs, 135Hz, 3.3mA | 2-C+, 90μs, 135Hz, 3.1mA |
| 6 | M | 74 | ET-plus | Left VIM | 3-C+, 140μs, 150Hz, 4.4mA | 3-C+, 140μs, 150Hz, 4.4mA |
| 7 | M | 67 | ET | Left VIM | 3-C+, 60μs, 130Hz, 2.5mA | 3-C+, 60μs, 130Hz, 2.5mA |
|  |  | 68 |  | Right VIM | 7-C+, 60μs, 130Hz, 4.3mA | Not Available |
| 8 | F | 57 | ET | Left VIM | 1-2+, 60μs, 130Hz, 1.8mA | 1-2+, 60μs, 130Hz, 1.8mA |
|  |  | 58 |  | Right VIM | 6-C+, 60μs, 130Hz, 2.3mA | Not Available |
| 9 | M | 65 | ET | Left VIM | 2-C+, 60μs, 130Hz, 2.4mA | Not Available |
| 10 | M | 79 | ET-plus | Left VIM | 2-C+, 60μs, 130Hz, 1.9mA | 2-C+, 60μs, 130Hz, 2.3mA |
| 11 | F | 55 | ET | Left VIM | 3-C+, 90μs, 130Hz, 4.0mA | Not Available |
| 12 | M | 64 | ET | Right VIM | 6-C+, 60μs, 130Hz, 2.1mA | 6-C+, 60μs, 130Hz, 2.6mA |
| 13 | M | 72 | ET-plus | Left VIM | 3-2+, 60μs, 135Hz, 3.2mA | 3-2+, 60μs, 135Hz, 3.9mA |
| 14 | M | 70 | ET-plus | Left VIM | 1-C+, 60μs, 130Hz, 2.4mA | 1-C+, 60μs, 130Hz, 2.4mA |
| 15 | M | 75 | ET-plus | Right VIM | 7-C+, 60μs, 135Hz, 3.0mA | 7-C+, 60μs, 135Hz, 3.1mA |
| 16 | M | 69 | ET | Left VIM | 3-C+, 60μs, 135Hz, 2.5mA | 3-C+, 60μs, 135Hz, 2.8mA |
| 17 | M | 60 | ET-plus | Left VIM | 3-C+, 60μs, 135Hz, 2.5mA | 3-C+, 60μs, 135Hz, 2.8mA |
| 18 | F | 63 | ET | Left VIM | 2-C+, 60μs, 145Hz, 3.0mA | 2-C+, 60μs, 145Hz, 3.0mA |
|  |  | 64 |  | Right VIM | 6-C+, 60μs, 130Hz, 2.3mA | 6-C+, 60μs, 130Hz, 2.3mA |
| 19 | F | 75 | ET-plus | Left VIM | 1-3-2+, 90μs, 160Hz, 2.6mA | 1-3-2+, 60μs, 160Hz, 2.8mA |
| 20 | M | 48 | ET | Left VIM | 3-C+, 90μs, 130Hz, 2.8mA | Not Available |
| 21 | F | 67 | ET-plus | Left VIM | 2-C+, 60μs, 135Hz, 2.1mA | 2-C+, 60μs, 135Hz, 2.1mA |
| 22 | M | 64 | ET-plus | Right VIM | 7-C+, 60μs, 135Hz, 1.5mA | 7-C+, 60μs, 135Hz, 1.7mA |
| 23 | M | 62 | ET | Right VIM | 7-C+, 60μs, 145Hz, 3.2mA | 7-C+, 60μs, 145Hz, 3.2mA |
| 24 | M | 70 | ET | Left VIM | 2-3-C+, 60μs, 135Hz, 2.5mA | 2-3-C+, 60μs, 145Hz, 2.5mA |
| 25 | F | 46 | ET-plus | Right VIM | 5-7+, 90μs, 145Hz, 3.8mA | Not Available |
| 26 | F | 64 | ET-plus | Left VIM | 2-C+, 60μs, 135Hz, 1.8mA | 2-C+, 60μs, 135Hz, 1.8mA |

**Supplementary Table 2.** Spearman’s correlation coefficients between FTM-B change from baseline at different follow-up times and described quantitative variables. *p-value < 0.05. (DBS = deep brain stimulation; FTM-B = Fahn-Tolosa-Marin Tremor Rating Scale-Part B; p = p-value; r = correlation coefficient; SF-36 = 36-Item Short Form Survey)

|  | | FTM-B change from baseline (treated limb) | | | |
| --- | --- | --- | --- | --- | --- |
|  | | Year 1 follow-up | Year 2 follow-up | Year 3-5 follow-up | Year 6-10 follow-up |
| Age at surgery | r | 0.11 | -0.45 | 0.00 | 0.27 |
|  | p | 0.66 | 0.06 | 0.99 | 0.56 |
| Age at tremor onset | r | -0.03 | 0.07 | 0.04 | 0.57 |
|  | p | 0.89 | 0.78 | 0.89 | 0.18 |
| Time from symptoms onset to surgery | r | 0.06 | -0.38 | -0.07 | -0.50 |
|  | p | 0.81 | 0.12 | 0.81 | 0.25 |
| Initial DBS voltage | r | 0.23 | 0.36 | 0.13 | -0.36 |
|  | p | 0.32 | 0.14 | 0.65 | 0.43 |
| Initial DBS pulse width | r | 0.13 | 0.24 | -0.18 | 0.18 |
|  | p | 0.59 | 0.35 | 0.52 | 0.70 |
| DBS frequency | r | 0.03 | 0.12 | -0.36 | -0.39 |
|  | p | 0.91 | 0.64 | 0.18 | 0.39 |
| FTM-B at baseline (treated limb) | r | **0.60** | **0.78** | 0.59 | 0.57 |
|  | p | **0.01*** | **<0.01*** | 0.29 | 0.30 |
| SF-36 at baseline | r | -0.06 | -0.14 | -0.43 | -0.77 |
|  | p | 0.80 | 0.57 | 0.11 | 0.07 |
| Beck Depression Inventory-II at baseline | r | 0.15 | 0.17 | 0.51 | 0.32 |
|  | p | 0.55 | 0.53 | 0.07 | 0.68 |

**Supplementary Table 3.** Point-biserial correlation between FTM-B change from baseline at different follow-ups and described quantitative variables. *p-value ≤ 0.05. (ET-plus = essential tremor plus; FTM-B = Fahn-Tolosa-Marin Tremor Rating Scale-Part B; MRI = magnetic resonance imaging; p = p-value; PD = Parkinson disease; r = correlation coefficient)

|  | | FTM-B change from baseline (treated limb) | | | |
| --- | --- | --- | --- | --- | --- |
|  |  | Year 1 follow-up | Year 2 follow-up | Years 3-5 follow-up | Years 6-10 follow-up |
| Gender | r | -0.28 | -0.24 | 0.02 | -0.21 |
|  | p | 0.22 | 0.34 | 0.94 | 0.65 |
| Family history of tremor | r | -0.15 | -0.11 | -0.21 | -0.56 |
|  | p | 0.52 | 0.66 | 0.45 | 0.19 |
| Family history of PD | r | **-0.44** | -0.46 | -0.18 | -0.67 |
|  | p | **0.04*** | 0.06 | 0.51 | 0.10 |
| MRI: Small vessel disease | r | -0.09 | -0.10 | 0.04 | -0.04 |
|  | P | 0.72 | 0.69 | 0.90 | 0.95 |
| MRI: Stroke | r | -0.27 | -0.22 | 0.03 | . |
|  | p | 0.26 | 0.40 | 0.91 | . |
| MRI: Other abnormalities | r | 0.19 | 0.01 | 0.09 | . |
|  | p | 0.44 | 0.97 | 0.77 | . |
| Handedness | r | 0.20 | 0.30 | 0.24 | 0.58 |
|  | p | 0.40 | 0.22 | 0.38 | 0.17 |
| Tremor in both hands at baseline | r | -0.17 | -0.14 | . | . |
|  | p | 0.46 | 0.59 | . | . |
| Tremor in one hand at baseline | r | 0.17 | 0.14 | . | . |
|  | p | 0.46 | 0.59 | . | . |
| Tremor in both lower limbs | r | -0.13 | -0.20 | 0.01 | 0.22 |
|  | p | 0.59 | 0.42 | 0.96 | 0.64 |
| Tremor in one lower limb | r | -0.13 | -0.22 | . | . |
|  | p | 0.59 | 0.38 | . | . |
| Voice tremor at baseline | r | -0.34 | -0.41 | **-0.60** | -0.62 |
|  | p | 0.14 | 0.09 | **0.02*** | 0.14 |
| Neck tremor at baseline | r | -0.31 | -0.44 | -0.39 | -0.33 |
|  | p | 0.19 | 0.07 | 0.16 | 0.46 |
| Facial tremor at baseline | r | -0.19 | -0.29 | -0.04 | 0.22 |
|  | p | 0.41 | 0.25 | 0.88 | 0.64 |
| Segmental (vs. generalized) | r | -0.08 | 0.06 | -0.30 | -0.22 |
|  | p | 0.75 | 0.80 | 0.28 | 0.64 |
| ET-plus | r | -0.10 | -0.30 | -0.39 | 0.12 |
|  | p | 0.67 | 0.22 | 0.15 | 0.80 |
| Response to alcohol | r | 0.24 | 0.28 | 0.20 | 0.53 |
|  | p | 0.31 | 0.25 | 0.47 | 0.22 |
| Response to propranolol | r | 0.00 | 0.01 | 0.17 | 0.29 |
|  | p | 0.99 | 0.96 | 0.54 | 0.53 |
| Response to primidone | r | -0.27 | -0.35 | -0.42 | -0.57 |
|  | p | 0.24 | 0.15 | 0.12 | 0.18 |
| Response to topiramate | r | 0.17 | 0.18 | 0.03 | 0.06 |
|  | p | 0.47 | 0.48 | 0.90 | 0.91 |
| Response to benzodiazepines | r | -0.24 | -0.08 | -0.24 | . |
|  | p | 0.31 | 0.75 | 0.39 | . |
